# Behavioral Microbiomics: A Multi-Dimensional Approach to Microbial Influence on Behavior

**DOI:** 10.3389/fmicb.2015.01359

**Published:** 2015-11-27

**Authors:** Adam C.-N. Wong, Andrew Holmes, Fleur Ponton, Mathieu Lihoreau, Kenneth Wilson, David Raubenheimer, Stephen J. Simpson

**Affiliations:** ^1^Charles Perkins Centre, The University of Sydney, Sydney, NSW, Australia; ^2^School of Biological Sciences, The University of Sydney, Sydney, NSW, Australia; ^3^Division of Infectious Diseases, Boston Children’s Hospital, Harvard Medical School, Boston, MA, USA; ^4^School of Molecular Bioscience, The University of Sydney, Sydney, NSW, Australia; ^5^Department of Biological Sciences, Macquarie University, Sydney, NSW, Australia; ^6^Centre National de la Recherche Scientifique, Centre de Recherches sur la Cognition Animale, Toulouse, France; ^7^Université Paul Sabatier, Centre de Recherches sur la Cognition Animale, Toulouse, France; ^8^Lancaster Environment Centre, Lancaster University, Lancaster, UK

**Keywords:** behavior, holobiont, microbiome, symbiosis, nutrition

## Abstract

The role of microbes as a part of animal systems has historically been an under-appreciated aspect of animal life histories. Recently, evidence has emerged that microbes have wide-ranging influences on animal behavior. Elucidating the complex relationships between host–microbe interactions and behavior requires an expanded ecological perspective, involving the host, the microbiome and the environment; which, in combination, is termed the holobiont. We begin by seeking insights from the literature on host–parasite interactions, then expand to consider networks of interactions between members of the microbial community. A central aspect of the environment is host nutrition. We describe how interactions between the nutrient environment, the metabolic and behavioral responses of the host and the microbiome can be studied using an integrative framework called nutritional geometry, which integrates and maps multiple aspects of the host and microbial response in multidimensional nutrient intake spaces.

## Introduction

Behaviors mediate the relationship between an animal and its changing environment, both abiotic and biotic. A key component of the biotic environment is the vast number of microbial species that live within and upon animals, many of which reside within the gut (the gut microbiome). An oft-neglected feature is the tension and disparity in biological scales between the host and its symbionts: the animal cells dominate the biomass, are typically isogenic (but phenotypically differentiated) and many have slow turnover; whilst the microbial cells are of lower biomass, up to orders of magnitude greater in numbers, genetically-diverse and have faster turnover (Whitman et al., 1998; Ley et al., 2006; Grice et al., 2008). Collectively, the evolutionary processes differ between the host and symbiotic partners, and understanding phenotypic changes of animal systems requires a perspective that encompasses microbial ecology. Fundamental physiological processes including tissue development (Stappenbeck et al., 2002), nutrient absorption (Gibson et al., 2004; Turnbaugh et al., 2006), immunity (Round and Mazmanian, 2009; Maynard et al., 2012), and circadian regulation (Thaiss et al., 2014; Zarrinpar et al., 2014; Leone et al., 2015) are emergent properties of the interactions between the host and microbiome. Mounting evidence now points to behavior, both individual and social (Heijtza et al., 2011; Cryan and Dinan, 2012; Ezenwa et al., 2012; Dance, 2014), as also reflecting inputs from the microbiome.

In this perspective article we explore animal behavior from the view of animals as communities of organisms in a complex environment that underlies the concepts of “holobionts” (Rohwer et al., 2002; Reshef et al., 2006). We propose “behavioral microbiomics” as a new field that emphasizes the multi-dimensional environment and its impact on host–microbe interactions and behavioral outcome. Using nutrition as an example, we introduce an integrative framework—nutritional geometry- and discuss its research and practical applications.

## Evolution of Behavior: Insights From Host–Parasite Interactions

Host–parasite dynamics offer a number of examples of how behavioral traits of the holobiont can be shaped by the interplay between the evolutionary interests of the different parties, leading to outcomes that may not favor the host (Adamo, 2013; Moore, 2013). One such example is the protozoan *Toxoplasma gondii*, which acts upon the host rodent’s amygdala to reverse the innate response to cat odors from repulsion to attraction, thus increasing the rodent’s predation risk while facilitating the parasite’s dispersal (Berdoy et al., 2000; Vyas et al., 2007; Hari Dass and Vyas, 2014). Other examples include entomopathogenic *Ophiocordyceps* fungi that “zombify” infected ants and trigger host leaf climbing, resulting in improved dispersal of fungal spores (Hughes et al., 2011) and salt marsh trematodes *Microphallus papillorobustus* that alter a range of behaviors of infected gammarids, making the host more vulnerable to predation by aquatic birds (Helluy, 1983). In addition, parasites can modify host reproductive behaviors (e.g., egg production), resulting in resource allocation shifts to favor the parasite rather than host fecundity (Lafferty and Kuris, 2009).

Host–parasite dynamics also covers examples of how the host can retaliate behaviorally. Changes in feeding behavior are considered a common host adaptive response to parasitism. These include self-medication by consuming protective plant secondary compounds to control or eliminate the parasites (Lozano, 1998; Raubenheimer and Simpson, 2009; Singer et al., 2009) and adjusting the intake of macronutrients to enhance immunity and/or to compensate for the infection costs (Lee et al., 2006; Ponton et al., 2011; Mason et al., 2014; Povey et al., 2014). Behaviors that operate at the group level are observed in social animals, such as “social immunity” (Cremer et al., 2007; Cotter et al., 2010). Examples include group-level behavioral thermoregulation, as seen in honeybees (*Apis mellifera*) producing a social fever against the fungus *Ascosphaera apis* (Starks et al., 2000), as well as other transmission-limiting behaviors such as grooming, detection and disposal of infected nest mates in social insect colonies (Reber et al., 2011). Social withdrawal associated with parasitism may also constitute an altruistic trait to limit the risk of infection to kin in the worker ants *Temnothorax unifasciatus* (Heinze and Walter, 2010).

**FIGURE 1:**
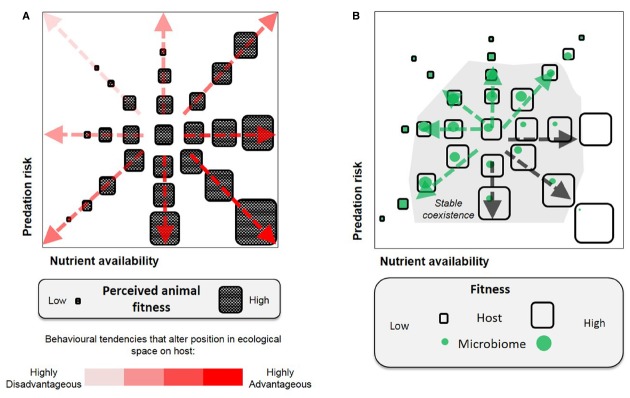
**Selection of animal behavior, illustrated by two dimensions of fitness parameters (nutrient availability and predation risk). (A)** A host-centric view, only behavioral traits advantageous to animal fitness would be selected for. **(B)** A holobiont view, behavioral selection is bounded by host–microbe associations and involves positive and negative feedbacks between the host and microbes [relatively advantageous to microbes (green arrows) or the host (black arrows)]. The host–microbe relationship is not intrinsically parasitic or mutualistic, but is defined by the fitness outcome imposed by the environmental context.

The above examples are readily reconciled with existing selection models for parasitism and mutualism (Sachs et al., 2011). Host–parasite interactions are typified as an antagonistic arms race in which hosts evolve to select against the common parasite genotypes (negative frequency-dependent) and this drives rapid parasite evolution (Dawkins and Krebs, 1979). By contrast, in mutualism, the host evolutionary interests are best served by maintaining the common symbiont genotypes (positive frequency-dependent), with the associations stabilized by mechanisms that promote partner choice and fidelity (Kaltenpoth et al., 2014). Though limited to binary host–microbe interactions, these models and examples demonstrate that holobiont behaviors can be driven by parallel and conflicting interests between the host and its symbiont (illustrated in Figure 1B). Furthermore, the adaptive or selective effects can be realized at the scale of an individual or a population.

## Behavioral Selection in the Multi-Partner Holobiont

In comparison to the host–parasite dynamics, understanding selection of behavior within the holobiont requires consideration of two further issues. First, many emergent properties of the microbiome involve multiple species and genotypes, with host behaviors reflecting microbiome composition at the level of community rather than individual microbial taxa. An example is the production of specialized fecal pellets (pap) by mother koalas. The pap is fed to the juvenile, providing it with a cocktail of microbes that confer both protection against toxins and the capability to extract nutrients from eucalypt leaves (Osawa et al., 1993). Parallel cases of microbiome sharing have been shown in social insects, such as trophallaxis in bees and termites (Koch and Schmid-Hempel, 2011).

Second, there are important ecological interactions and asymmetries within the members of the microbiome, such that the optimal response to a given external environment is not the same for all members, or for the host (Figure 1B). A related point is that the same microbial taxa may have differing net impacts on host fitness according to environmental circumstances. For example, paramecia infected with the bacterium *Caedibacter* suffer reduced reproductive rate, but outcompete uninfected paramecia when in mixed culture through the release of a toxin from the resident bacterium (Kusch et al., 2002). The endosymbiotic bacterium, *Wolbachia*, known for manipulating host reproductive traits including behavior to facilitate its transmission, has been shown to protect the *Drosophila* host from virus infection (Hedges et al., 2008). In humans, colonization of *Helicobacter pylori* in the stomach is a major risk factor for peptic ulcer disease and gastric cancer (Forman et al., 1993), yet human population studies and experiments on mice suggest the bacterium may protect the hosts against certain autoimmune diseases, such as asthma (Chen and Blaser, 2008; Arnold et al., 2011).

Because the optimal response to an external environmental change is not the same for all holobiont members, when a host attempts to change behavior in response to environment other holobiont members may resist this change. While a host can alter the selective environment for the microbiome through its behavior, or evolve mechanisms such as sanction-and-reward to enforce microbial compliance (see review: Douglas, 2008), the same applies to microbiome members that benefit by influencing holobiont behavior under environmental selection. These benefits include sustaining associations with or even dominating the host. Taking microbiome transmission as an example, continuous sharing of microbiome is a prevalent feature in some mammals and social insects. The evolution of such social behavior has been widely suggested a host strategy against harmful infections (Koch and Schmid-Hempel, 2011); but it also serves the interests, and could be driven by the microbiome as it sustains microbial access to the nutrient-rich intestinal habitat.

## Modifications of Behavior by Microbes- or Host-Adaptive Traits?

We next consider two examples to illustrate how behavior can reflect feedbacks arising from both host and microbiome.

### Feeding Behavior

**FIGURE 2:**
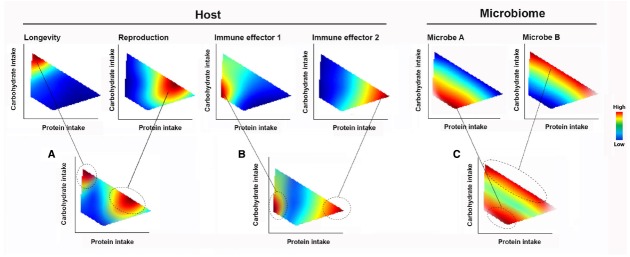
**The Geometric Framework of nutritional ecology (GF).** Complex nutrient environment is delineated into dimensions (for illustrative purpose, only two dimensions, proteins and carbohydrate intakes are shown). The performance responses of holobiont components to nutrient intake are displayed as heatmap landscapes, with the strength of response indicated by a color code (warmer colors show strongest responses). Changes in nutrient intake allow a host navigating the trade-off spectrum in the nutrient environment. Animal **(A)** longevity, reproduction and **(B)** immune effector responses are all dependent on the balance of nutrient intake. The same tradeoffs also apply to **(C)** different members of the microbiome. Feeding behavior can be selected both in total amount of calories and/or specific amount of nutrient components.

When an animal makes feeding decisions, various internal and external stimuli are integrated into behavioral responses. Changes in feeding behavior allow an animal to navigate the spectrum of trade-offs for different physiological traits, such as longevity-reproduction and expression of immune effectors (Figures 2A,B). In the context of the holobiont, what a host eats changes the microbiome composition and, as a consequence, affects microbiome-derived signals that feedback onto immune and metabolic functions (Figure 2C). Host feeding can be influenced by these feedback signals, along with direct processes by the microbes including the provisioning of nutrients and competing for host-intake nutrients (Douglas, 2009, 2011; Nicholson et al., 2012; Kostic et al., 2013).

A feature of modern societies has been the rising prevalence of diseases related to unhealthy feeding habits (e.g., obesity, metabolic syndrome; Ng et al., 2014). In the traditional host-centric view (Figure 1A), these disease-causing behaviors are not readily explained as adaptive or selected since host health is compromised. In addition, many of the diseases have been associated with specific microbiome signatures, the basis for “dysbiosis” (Tilg and Kaser, 2011); and recent studies have demonstrated a direct link between attributes of the microbiome and animal food intake, e.g., axenic rats receiving microbiome from obese counterparts had elevated food consumption relative to microbiome from lean rats (Duca et al., 2014). These phenomena lead to a question: Might pathological feeding habits (e.g., anorexia or bulimia nervosa) arise as maladaptive behaviors selected for by interactions between the environment and components of the microbiome? We hypothesize that host dietary choices and feeding habits can be modified as a result of feedbacks to prioritize specific host–microbe interactions, on the basis of the holobiont concept that anticipates tradeoffs in fulfilling the requirements of different partners. Such responses may be driven toward stable co-existence of both partners subject to environmental selection (Figure 1B).

### Social Behavior

Microbial influence on social behaviors is an emerging area of study (Wang and Kasper, 2014). Some of these traits may be linked to pathological conditions (e.g., stress, autism). From an evolutionary perspective, an important question is whether social behaviors affected by the microbiome arise as microbial-selected or host-adaptive traits, or whether such a distinction exists when they serve the interests of both (or neither) of the partners.

As discussed above, some of these behavioral traits improve symbiont niche protection and transmission (Lombardo, 2008). For example, sharing of the microbiome via physical contacts has long been demonstrated in herbivores relying on cellulolytic gut symbionts to degrade plant materials (Troyer, 1984). Such behaviors have been interpreted as driven primarily by host requirements. However, new evidence is pointing toward expression of behaviors that can be considered manipulative by the microbes, though in some cases a clear benefit to the microbial community is difficult to envisage. For example, the stinkbug *Megacopta punctatissima* provides its offspring with microbes in capsules at egg deposition. When the microbes are experimentally depleted, normal aggregation behavior of the hatchlings is disrupted (Hosokawa et al., 2008). The distinct behavioral characteristics of two laboratory mouse strains, Balb/c (nervous, hesitant) and c Swiss (social, exploratory) appeared exchangeable through reciprocal microbiome transplantation between axenic and conventional mice (Bercik et al., 2011).

Other examples include social behaviors that are directly related to animal life histories, such as kin recognition, mate selection and aggregation. The microbiome may affect kin recognition and mating by direct release of microbial metabolites, and/or by changing the expression of host pheromones. For example, *Drosophila melanogaster* aggregation on food is associated with the presence of chemical signals from the gut microbiome among peers (Venu et al., 2014). The presence of microbes, as well as their community composition, can also affect *Drosophila* mating preference (Sharon et al., 2010; Lize et al., 2014). Another example is locust swarming, mediated in part by semiochemicals released in fecal pellets. These aggregation pheromones are produced by bacteria located in the locust hindgut (Dillon et al., 2000). It has been suggested that infection by a microsporidian parasite inhibits this behavior by suppressing these hindgut bacteria (Shi et al., 2014). The influence of microbiome on kin recognition has also been suggested in the spotted hyena (*Crocuta crocuta*), where members of different social groups carry distinguishable olfactory cues associated with different microbiome (Theis et al., 2012). In humans, the skin microbiome influences the scent of individuals in ways that reflect kinship (Kuhn and Natsch, 2009), with odor profile and preferences affected by the host major histocompatibility complex (MHC) genes (Penn and Potts, 1998; Havlicek and Roberts, 2009).

## Mechanisms of Microbial Impact on Animal Behavior

Neuromodulatory compounds within the host nervous system are also released or sensed by different types of microbes, raising the possibility that microbes directly manipulate behavior through common chemical messengers, and that certain neurological pathways may be susceptible to microbial feedbacks. Different types of microbes have been shown to impact host behavior directly through small molecules acting upon neuro-endocrine circuits, and indirectly by influencing host epigenetic state and immune functions (see reviews Adamo, 2013; Hemarajata and Versalovic, 2013; Wang and Kasper, 2014).

Considerable interest has been focused on the “gut-brain axis” (Cryan and Dinan, 2012). For example, there is a rich array of G-protein-coupled receptors (GPCR) in the gut connected to neuroendocrine circuits. A number of such receptors are dedicated to sensing microbial metabolites (Tan et al., 2014) and others have been mapped to neurons that can trigger specific behaviors (Sakurai et al., 1998; Alexander et al., 2009). Some of the modulators influenced by the microbiome include bile acids, short chain fatty acids (SCFAs) and gut peptide hormones associated with feeding and energy regulation (Samuel et al., 2008), as well as neurotransmitters (e.g., GABA) that can be produced by microbes such as *Lactobacillus* and *Bifidobacterium* (Barrett et al., 2012). Some animal behavioral changes resulting from microbial intervention have been attributed to single metabolites, such as 4-ethylphenyl sulfate (4EPS) in mice (Hsiao et al., 2013). A key feature is that an animal’s behavioral response is expected to be dose-dependent to microbial metabolites that are regulated by microbial activities as well as exposure to specific sites of receptors, both of which are tightly linked to nutrition.

## Behavioral Microbiomics: A Nutritional Ecological Perspective

Behavioral microbiomics aims to provide an integrative understanding of how behavior reflects host–microbe interactions within a given environment. Of all aspects of environment, nutrition is the most fundamental in shaping the responses of the holobiont system. Essentially, the nutritional resources provided to the microbiome are dependent on host feeding behavior and host secretions. Once food is consumed, the composition and physical form of the ingesta changes as it passes down the gastrointestinal tract, offering microbes at different locations a changing complement of nutrients. Not only does the host require multiple nutrients in appropriate quantities and balance to perform optimally (Simpson et al., 2015), each member of the microbiome has its own multidimensional nutritional target—which may differ from that of other members of the microbiome and the host (Simpson and Raubenheimer, 2012). In turn, the host may derive essential nutrients from members of the microbiome when it lacks the metabolic capacity to gain these *de novo* from the diet (Douglas, 2011; LeBlanc et al., 2013).

To understand the interactive effects of nutrients and the microbiome on animal behavioral outcomes, what is required is a means of encompassing the holobiont within a unifying nutritional framework that takes account of multiple dimensions of nutrients, host responses, and the diversity of responses among the members of the microbial community. Yet, many studies to date have only considered foods as uniform commodities, collapsing such into a single dimension, or only focused on varying single components when studying the relationships between diet, microbiome and animal physiology (Crawford et al., 2009; Daniel et al., 2014; Palmnas et al., 2014). Recent advances in nutrition research have provided an integrative approach, known as the geometric framework (GF) or nutritional geometry (NG; Simpson and Raubenheimer, 2012), which explicitly takes account of the interactions among nutrients. In this approach, animals are confined to a series of diets differing systematically in multiple nutrient dimensions (typically the ratios and concentrations of individual macronutrients, but any nutrient can be included), and then host and microbial responses are mapped as topological surfaces in multidimensional nutrient intake space constructed *in silico* using R (library “fields”; Furrer et al., 2007). It has been used to define and associate relationships between life histories, growth, metabolic, immune, and behavioral responses in organisms from slime molds to insects and humans (Lee et al., 2008; Dussutour et al., 2010; Gosby et al., 2011; Solon-Biet et al., 2014). For example, a recent NG mice study compared food intake, lifespan, reproduction, cardio-metabolic health, immune status of mice fed *ad libitum* in one of thirty diets varying in ten different protein to carbohydrate to fat ratios (P:C:F; Solon-Biet et al., 2014). The data have been used to construct a 3D macronutrient space and quantify the significance of total and relative macronutrient content in diet on mice feeding behavior and health outcomes, such as to provide new understanding on the nutritional basis that underlies the longevity-promoting effect of dietary restriction. Changes in the abundance of a symbiont have also been mapped to nutrient intake spaces in *Drosophila* (Ponton et al., 2015). Hence, NG offers the means to delineate relationships between components of the holobiont (individual microbial taxa, microbial community structure, host physiology, and behavior) and nutrition, and to generate causal hypotheses that can be explored experimentally using gnotobiotic animals, drugs, experimental manipulations, and genetic models. Additionally, recent developments using agent-based modeling approaches have begun to describe how these complex interactions can scale up at different levels of biological organizations, in groups, societies, populations, and communities of organisms (Simpson et al., 2010; Lihoreau et al., 2015).

Figure 2 shows a simplified hypothetical GF model involving two macronutrient dimensions. If longevity is its primary fitness concern, an animal would be expected to maximize carbohydrate intake at low intakes of protein (Figure 2A). However, other fitness traits such as reproduction require higher protein intakes. The relative importance of promoting different host fitness traits depends on the environment and stage in the life course (Figures 2A,B). An animal’s feeding choices also shape the competitive dynamics among microbes and their interactions with the host—as illustrated using two members (A and B) of the hypothetical microbial community (Figure 2C). The landscapes for each member describe what is termed their “realized nutritional niche” (Kearney et al., 2010). Considering the case of diets defined by fixed carbohydrate content, it can be seen that there is a range of possible outcomes for A and B, depending on the host’s protein intake (Figure 2C, indicated by a gray dashed line). Thus, the response of gut microbiomes that consist of diverse species with different nutritional niches and the associated impacts on host physiology and behavior can only be understood by investigating multiple nutrient dimensions. As critical dimensions of the holobiont response and nutrient environment are revealed using NG, it becomes possible to predict, test and couple host nutritional behaviors with systematic variation of the microbiome.

## Implications for Health

From a clinical perspective, behavioral microbiomics offers new opportunities for disease diagnosis, prevention and intervention.

### Diagnosis

There is the potential for exploiting the microbiome as a biomarker to assess the physiological context of psychological state and behavioral tendencies of an individual. Studies showing correlative microbiome compositional changes in metabolic and behavioral disorders have contributed to the concept of “dysbiosis” (Hawrelak and Myers, 2004). Generally, “dysbiosis” refers to a microbiome state (or composition) that is associated with host pathological conditions. To address the relationship between dysbiosis and behavioral disorders, we can adopt insights from behavioral genetics, such as using genome-wide association studies (GWAS) to establish links between genes (microbiome) and specific behavioral traits (Psychiatric et al., 2009).

### Prevention and Intervention

Changes in the environment can lead to a disease state. A prerequisite to develop microbial approaches to prevent or treat disorders associated with dysbiosis is identifying which dimensions of environment are promoting such vulnerability, and the effector host–microbe interactions that maintain the disease state. Preclinical trials have already shed light on direct impact of single probiotics on animals’ psychological state and social behaviors. For example, hyper-responsiveness of axenic mice to stress was reversed by re-association with a native human gut bacterium, *Bifidobacterium infantis* (Sudo et al., 2005). Chronic treatment of *Lactobacillus rhamnosus* also reduced anxiety- and depression-related behavior in mice, linked to reduced corticosterone and altered spatial expression of GABA receptors (Bravo et al., 2011). Administration of *Bacteroides fragilis* appeared to alleviate many behavioral symptoms of autism spectrum disorder in axenic mice, associated with changed serum metabolites and restored gut permeability (Hsiao et al., 2013). Thus, the potentials exist to develop targeted dietary, pre/pro/anti-biotic and pharmacological interventions that take environmental influence into considerations.

## Concluding Remarks

An important role of animal behavior is to provide a means of regulating an animal’s physiological state through adjusting interactions with the environment. Behavior also serves to regulate its microbial component and the relationships between animal behavior and microbiome are reciprocal. Through acknowledging the complexity of the environment, behavioral microbiomics encourages looking at the influence of microbiome on animal physiology through the prism of behavior in multiple environmental dimensions. Here, we provide a NG framework to study how the different aspects of the environment can modulate the host-microbiome interaction network. Delineation of these drivers will provide ecological and mechanistic understanding on microbiome effects on animal behaviors and behaviorally-related diseases.

### Conflict of Interest Statement

The authors declare that the research was conducted in the absence of any commercial or financial relationships that could be construed as a potential conflict of interest.
